# Self-management needs of Irish adolescents with Juvenile Idiopathic Arthritis (JIA): how can a Canadian web-based programme meet these needs?

**DOI:** 10.1186/s12969-018-0287-0

**Published:** 2018-11-08

**Authors:** Grace O’Sullivan, Siobhán O’Higgins, Line Caes, Sophia Saetes, Brian E. McGuire, Jennifer Stinson

**Affiliations:** 1 Centre for Pain Research; School of Psychology, College of Arts, Social Sciences & Celtic Studies, Galway, NUI Ireland; 2 Division of Psychology; Faculty of Natural Sciences, University of Stirling, Stirling, Scotland, UK; 3 The Hospital for Sick Children, Toronto, Canada; 4 Child Health Evaluative Sciences, The Hospital for Sick Children, Toronto, Canada; 5 Lawrence S. Bloomberg Faculty of Nursing, University of Toronto, Toronto, Canada

**Keywords:** Juvenile arthritis, Adolescence, Self-management, Online intervention, Needs assessment

## Abstract

**Background:**

*Juvenile Idiopathic Arthritis* (JIA) affects over 1000 children and adolescents in Ireland, potentially impacting health-related quality-of-life. Accessible self-management strategies, including Internet-based interventions, can support adolescents in Ireland where specialist rheumatology care is geographically-centralised within the capital city. This study interviewed adolescents with JIA, their parents, and healthcare professionals to (i) explore the self-management needs of Irish adolescents; and (ii) evaluate the acceptability of an adapted version of a Canadian JIA self-management programme (*Teens Taking Charge: Managing Arthritis Online,* or *TTC*) for Irish users.

**Methods:**

Focus groups and interviews were conducted with Irish adolescents with JIA (*N* = 16), their parents (*N* = 13), and Irish paediatric healthcare professionals (HCPs; *N* = 22). Adolescents were aged 12–18 (M_age_ = 14.19 years), and predominantly female (62.5%). Participants identified the needs of adolescents with JIA and evaluated the usefulness of the TTC programme. Data were analysed using a thematic analysis approach.

**Results:**

Five themes emerged: *independent self-management; acquiring skills and knowledge to manage JIA; unique challenges of JIA in Ireland; views on web-based interventions;* and *understanding through social support*. Adolescents acknowledged the need for independent self-management and gradually took additional responsibilities to achieve this goal. However, they felt they lacked information to manage their condition independently. Parents and adolescents emphasised the need for social support and felt a peer-support scheme could provide additional benefit to adolescents if integrated within the *TTC* programme. All participants endorsed the *TTC* programme to gain knowledge about JIA and offered suggestions to make the programme relevant to Irish users.

**Conclusions:**

There is scope for providing easily-accessible, accurate information to Irish families with JIA. The acceptability of adapting an existing JIA self-management intervention for Irish users was confirmed.

## Introduction

Juvenile Idiopathic Arthritis (JIA) describes a cluster of exclusionary rheumatic diagnoses “…*that begin before the age of 16 years, persist for more than six weeks, and are of unknown origin*” [1–4]. JIA is the most common childhood rheumatic disease; in the Republic of Ireland, approximately 1200 children have JIA and over 100 children are diagnosed annually [5]. Symptoms include joint pain, swelling, inflammation, stiffness and fatigue, leaving children in significant pain and making school attendance or daily tasks difficult [6]. Physical and emotional symptoms restrict social interactions for these adolescents, isolating them from their peers, and has the potential to negatively impact health-related quality-of-life (HRQL) [7–11]. Compared to healthy peers, adolescents with JIA often report decreased HRQL and scores deteriorate with increased disease severity, pain, and degree of disability [8, 9, 12]. However, children with newly-diagnosed JIA can achieve HRQL similar to healthy children [13, 14], provided intervention begins at diagnosis.

Treatment for JIA is often multidisciplinary and includes pharmacological treatments, physiotherapy and psychological support. In the absence of a cure, treatments prioritise secondary prevention of complications and improving HRQL through disease self-management of *“physical or psychological consequences, and lifestyle changes*” [15]. Greater awareness and self-management among adolescents reduces associated adverse health outcomes [16]. Adolescence is a crucial life-stage for young people to gain independence from parents and take responsibility for their own needs. This applies to every adolescent, and particularly to those with chronic diseases, who must gradually learn independent disease self-management, and navigating the healthcare system. However, many adolescents with JIA do not receive comprehensive JIA self-management guidance [17–20], and feel unprepared to transfer from paediatric to adult care [8, 19, 21]. Additional barriers for effective self-management are perceived within the Irish health-care context, and raised by the participants of this study: long wait-times for appointments, inconsistent access to services outside of urban areas, and limited availability of child and adolescent rheumatology professionals; the latter becoming common across Europe, with many countries failing to supply satisfactory training in adolescent health and medicine [22, 23]. Accessible interventions to assist adolescents with self-management strategies can reduce these barriers [24–28].

Tailored online interventions can improve behavioural outcomes [28–30], and a Canadian study of adolescents with JIA, their parents and healthcare professionals revealed that adolescents wanted to manage JIA to maintain a ‘normal’ lifestyle with peers [22]. This prompted the development, usability and feasibility-testing of ‘*Teens Taking Charge*’ (TTC), a self-management website for adolescents with JIA [22]. *TTC* contains twelve weekly modules with themed lessons and videos for adolescents, including skills-training, communication, advocacy, information on treatments, and service availability (e.g., school assistance). A parent site contains the same information without the twelve-week structure, and two additional modules on promoting their child’s independence. Studies implementing *TTC* reported improvements in disease-related knowledge, decreased pain, and increased exercise adherence [12, 27, 29]. Before implementing programmes in other health-care contexts, it is imperative to evaluate their cultural appropriateness in the target population, who may experience different issues to adolescents elsewhere [30]. As a result, hypotheses were not specified at the outset. The primary aims were to (i) conduct a qualitative exploration of the self-management needs of Irish adolescents living with JIA, from their own perspective, and of their parents and healthcare professionals; and (ii) evaluate whether the *TTC* self-management website could be successfully adapted to meet the needs of Irish users.

To achieve this, qualitative interviews and focus groups were conducted with Irish adolescents with JIA, their parents, and paediatric health-care professionals.

## Methods

### Participants

All potential participants were identified through partnership with [1] the paediatric rheumatology departments of two large specialist children’s hospitals in Dublin, Ireland, and [2] Irish arthritis organisations, *iCAN (Irish Children’s Arthritis Network)* and *Arthritis Ireland,* who identified local primary care physicians, and families which met the eligibility criteria. This ensured the involvement of healthcare professionals (HCPs) from the core rheumatology teams within the children’s hospitals and from primary care centres across Ireland. For adolescents and parents, eligibility screening was completed via telephone, using a pre-defined script. Eligibility criteria were: (i) adolescent is 12–18 years old, (ii) has diagnosis of a JIA subtype classified by the International League of Associations of Rheumatology (ILAR) [31], and (iii) has no comorbid conditions. Following inclusion criteria from similar studies, we used convenience sampling [22]. We did not differentiate between JIA subtypes, in line with the study’s primary aim to qualitatively explore the self-management needs for adolescents with JIA.

### Procedure

Once participation was agreed, dates for the adolescent and parent focus groups were arranged in three Irish cities closest to participating families, to allow participants to attend a conveniently-located focus group. Those unable to attend any focus group were offered an individual interview (in person, or via telephone; Table 1).

**Table 1:** – Participant breakdown by group, location, and type of participation

	Focus group			Individual interview		Total
	Galway	Cork	Dublin	In person	Phone interview	
Adolescents	4	2	4	1	1	16^a^
Parents	4	3	4	1	1	13
HCPs	5	–	12	5	–	22

^a^16 adolescents were involved, as two parents participated without their children (1 in Cork; 1 phone interview)

Adolescents, parents, and HCPs were interviewed in their respective groups by an interviewer and assistant. Focus groups lasted 60–75 min; individual interviews lasted 35–50 min. All interviews explored the challenges of living with (or treating those with) JIA, knowledge of medication or other therapies, how adolescents manage their condition, and sources of information [interview guides were adapted from previous needs assessments [22]]. The final section focused on the features and usability of the *TTC* programme for Irish families. This generally lasted 10–20 min, with adolescents spending more time in discussion than parents or HCPs. To facilitate this, all participants received access to the *TTC* website two weeks beforehand, and the website was also displayed on-screen during the interview.

### Data analysis

Demographic data were analysed using descriptive tests in SPSS 22.0. Interviews were digitally-recorded and transcribed verbatim (GOS; SS). Transcriptions were analysed independently by two of the authors (GOS; SOH), using NVivo.11 [32], to compare to previously-identified themes [22] and identify potential new themes on the impact of JIA on daily life and on the future. New themes were agreed by both authors, who analysed transcriptions a second time against new and existing themes for confirmation.

## Results

**Fig. 1:**
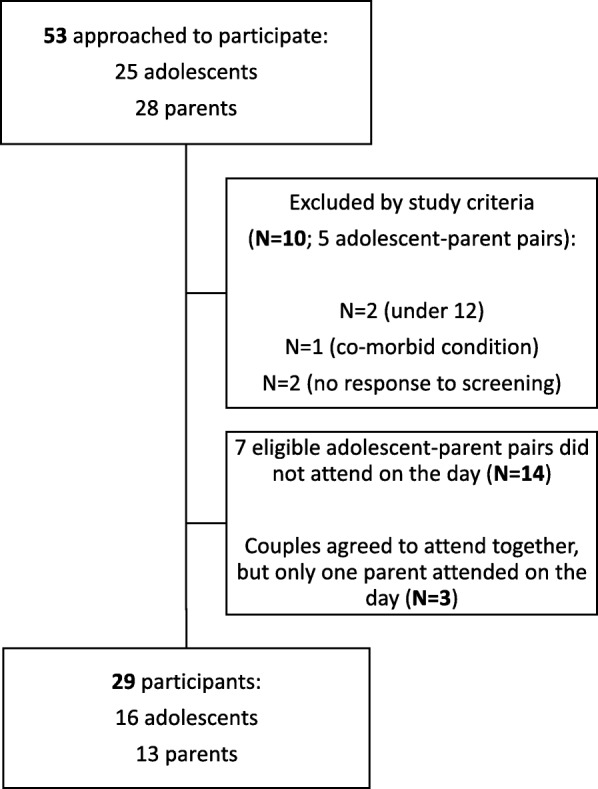
– Screening process for parent and adolescent participants

Fifty-three parents and adolescents were approached to participate (25 adolescents; 28 parents), with 43 meeting eligibility requirements. However, 14 of these eligible respondents (7 parent-adolescent pairs) did not attend their focus group, leaving 29 participants who met eligibility requirements (16 adolescents, 13 parents) (Fig. 1).

The participating adolescents were mostly in early (11–14 years, *N* = 8) or mid adolescence (15–16 years, *N* = 7), with one late-stage adolescent (17–18 years; *N* = 1) (M = 14.19 years; SD = 2.07). Most were female (*N* = 10; 62.5%) and of Irish nationality (*N* = 15; 93.7%). Mean time since diagnosis was 3.6 years (SD = 3.28 years; range = 0.58–13.83 years) and the most frequent subtype was psoriatic arthritis (*N* = 5; 31.3%) (Table 2).

**Table 2:** – JIA subtypes within the adolescent group

Arthritis subtype	Number in sample
Systemic	2
Polyarticular (RF Negative)	1
Polyarticular (RF Positive)	2
Psoriatic	5
Enthesitis-related	4
Other	2 (1 Poly/systemic overlapping; 1 undifferentiated)
Oligoarticular	0
Extended oligo-articular	0

Of the thirteen parents, most were biological mothers (*N* = 12; 92.3%), Irish (*N* = 12; 92.3%), and the typical age-range was 40–49 years (*N* = 10; 76.9%). Most were married (*N* = 10; 76.9%), had a university degree (*N* = 7; 53.9%), and were employed full-time or part-time (*N* = 8; 61.5%). Two were full-time carers for their child (*N* = 2; 15.4%).

Eligibility for HCPs included minimum one year of experience in working with JIA. All but two of the approached HCPs participated in the study. The 22 HCPs had an average of 8.45 years of experience working with chronic pain and were representative of most disciplines involved in treating adolescents with JIA **(**Table 3**).**

**Table 3:** – Overview of the HCP group

Profession	Frequency	Percentage (%)
Physician	5	22.7
Nurse	5	22.7
Physiotherapist	5	22.7
Psychologist	1	4.5
Physical Therapist	1	4.5
Occupational Therapist	5	22.7
Demographics		
Mean age range	30–39 years (*N* = 10; 45.5%)	
Gender	Female (*N* = 16; 72.7%); Male (*N* = 6; 27.3%)	
Highest educational level	Bachelor’s degree (*N* = 8; 36.4%) Master’s degree (*N* = 5; 22.7%) PhD or Medical degree (*N* = 6; 27.2%) Other (*N* = 3; 13.6%)	
Mean years of clinical experience	16.8 years (SD = 9.79 years; range = 3–46)	
Mean years of experience with chronic pain	8.45 years (SD = 5.47 years; range = 0–16)	
Work in a multi-disciplinary pain team?	Yes = 10 (45.5%)	

### Thematic clusters

Across all participant groups (parents, adolescents, HCPs), four main themes emerged: [1] *independent self-management (*including the sub-theme *“the impact of JIA on the future”* for parents and adolescents only); [2] *acquiring JIA knowledge and skills;* [3] *the unique challenges of JIA in Ireland;* [4] *web-based approaches to self-management*. A fifth theme appeared from parent and adolescent accounts: *experiencing understanding through social support*. Each theme and subtheme are discussed in detail below.

#### 1. Independent self-management

Most adolescents wanted to become more self-sufficient before transferring to adult care. Some adolescents started this process by attending appointments alone:

*Mam takes care of hospital appointments and bloods. I take care of my injections…She tries not to remind me anymore, I have to learn one day* (Adolescent 7)

Parents of younger adolescents encouraged responsibility over a gradual period:

*She’s twelve, but I get her into some OT [occupational therapy] appointments on her own, and I meet up with them after* (Parent 4)

*She does her injections all the time now. We’d be there with her though* (Parent 13)

Medication presented a major barrier to gaining independence for adolescents, with parents often reminding them or administering it for them:

*I take two injections every week and I always forget. I’ll be in bed and Mum will shout up to me ‘Have you taken your medications?’ And I’m like ‘Oh!*’ (Adolescent 16).

One HCP felt that overprotective parental attitudes prevented adolescents from becoming self-sufficient:

*A lot of it is breaking away from their parents and accepting the responsibility. Teenagers think ‘Oh Mum or Dad will do that’. It’s difficult for the parents to trust them* (HCP 20)

The HCPs suggested building trust gradually, through increasing the responsibilities handled by the adolescents, and offering support during that transition:

*It’s giving them responsibility and saying, ‘You can do it, and if you need support or resources, here they are’* (HCP 20)

### Impact of JIA on the future

Adolescents had not considered a future with JIA beyond career concerns (understandable given the younger age of this cohort). Parents had weighed the impact of JIA on their child’s future:

*I’ve always wanted to do teaching…But I’m worried I’m not going to be able to stand for six hours* (Adolescent 17)

*You just want them to go out and do the same things as their peers. Get the job, go to college. You want them to be normal, to be like everyone else* (Parent 19)

Generally, parents and adolescents prioritised managing JIA day-to-day, rather than planning for the future.

#### 2. Acquiring knowledge and skills to manage JIA

- ***Acquiring arthritis knowledge and awareness***

Adolescents gained most of their information on JIA from parents, which was limited as parents struggled to find accurate information themselves. Sources of information included medical pamphlets, websites and other parents, who often ‘crowdsourced’ their knowledge:

*I was quite young when I got it, so the doctors told my parents and my parents told me* (Adolescent 8)

*[Daughter] was getting more pains, and [another parent] said to me ‘If you’re not getting Omega-3’s, she’ll end up with more pains in her muscles’* (Parent 14)

Parents often used Google or Facebook pages of arthritis support organisations; however, such information was considered insufficient:

*When you’re online in American or Canadian stuff, they’re [medications] different names! They don’t know what we take, and they’ll be taking different things. You have to Google what it is*... (Parent 15)

Parents of newly-diagnosed children relied heavily on more experienced parents:

*I’m at such an early stage, I don’t know where to turn. If she’s having a flare, what do I do? I’m learning by the other parents* (Parent 18)

*This is something I’ve been going on about, there’s no information pack, you’re thrown into this... It’s like getting hit by a bus* (Parent 3)

However, HCPs emphasised that literature was readily available:

*When they come to physio they get a lot of paperwork about activity, lifestyle, balance, sleep, hygiene, and exercise programs* (HCP 22)

Though parents and HCPs disagreed over the availability of information, each agreed that a comprehensive source was needed for Irish families to learn JIA self-management skills.

b) ***Managing pain and discomfort***

All adolescents followed pharmacological treatment plans at the time of interview and many attended physiotherapists for joint pains. For most, managing pain and discomfort was achieved by reducing or switching activities:

*I’m very sporty. I did Irish dancing, boxing and hurling. But I had to give up all of them, I was too sore* (Adolescent 13).

For most adolescents, the primary motivation for managing pain and discomfort was to join in activities and appear ‘normal’ with friends:

*It can affect your friendships. If they’re going out and you come too, you can’t keep up. Or if you try, you need physio for the next three days* (Adolescent 35).

Parents and HCPs did not discuss pain and discomfort.

c) ***Managing emotions***

The language describing emotional struggles varied greatly between participant groups. Adolescents and HCPs discussed issues matter-of-factly, while parents used emotive language, highlighting the strain they felt:

*I would say* *fight,* *fight,* *fight* *for everything you can* (Parent 14)

*My daughter takes an injection every day. And the* *battle* *we have is every evening. It’s a huge* *battle* (Parent 19)

Formal emotional support for JIA diagnoses was hardly discussed, as only a few adolescents had attended appointments with a psychologist:

*I was in psychology for a bit, which I didn’t feel was needed. Even when I was doing it. It was good that even if I didn’t need it…it was available* (Adolescent 15).

Parents found day-to-day management of JIA stressful. They appreciated that psychological assessment could help their child, but accessibility issues were prevalent, confirmed by HCPs:

*The service is disjointed. [Area] have a physio unit and OT, but mental health services are in a different area. It’s difficult for people to get to* (Parent 34)

*We have no psychologist in the paediatric department and the only psychological help is from referring it through Child and Adolescent Mental Health Services and there’s waiting lists...* (HCP 11)

Adolescents and parents preferred informal support from peers instead of psychological services (see *Section 5: Gaining Understanding through Social Support*). HCPs theorised this came from negative attitudes in Ireland towards seeking psychological services:

*It’s seen that if you need psychology, you aren’t able to cope. It’s ‘What’s wrong with you?’* (HCP 26)

*If you see psychology as a treatment for pain, they automatically think ‘It’s in my head’ or ‘We don’t believe you’* (HCP 32)

Frustrated by the stigma and accessibility issues for psychologists, HCPs encouraged their adolescent patients to seek support from informal sources:

*I suppose the advice is to talk to a peer or a friend and maybe link into counselling, until they can get them in* (HCP 24)

#### 3. Unique challenges of a JIA diagnosis in Ireland

While elements of JIA diagnosis are similar worldwide, all participant groups perceived that Ireland had unique challenges, including lack of infrastructure, disproportionate division of services across the country, and cultural differences.

- ***Long wait times for diagnosis***

The primary challenge for Irish families was receiving a diagnosis. With only three paediatric rheumatologists (all located in the capital city), there were significant delays in initial appointments. All participant groups mentioned lengthy wait times for services:

*They wanted us to see [a psychologist] when I got diagnosed, which is nearly three years ago, and we’re still waiting to see one* (Adolescent 3)

*We opted to go privately. The wait time for Rheumatology was two years or more going the public route…* (Parent 34)

b) ***Unequal access to services***

This geographically-centralised care system involved long journeys for families outside Dublin, who missed work or school to travel for appointments. Some scheduled multiple appointments for one date, to reduce frequent cross-country trips, at the cost of long days:

*Yeah, early start. We’d get up at five in the morning, to be there for 8:30. And we wouldn’t get home until 7pm* (Adolescent 4)

All participant groups agreed that the geographical concentration disadvantaged families outside the capital:

*Our patients can be five hours from Dublin. We have a paediatrician who has an interest in rheumatology. He’ll give infusions so they don’t have to travel up and down* (HCP 20)

*I’m still struggling to get OT. I’d say if you were in Dublin it would be easier, but in [area] it’s very hard* (Parent 34)

c) ***High expenditure associated with the disease***

JIA placed a heavy financial burden on Irish families. Government-funded medical treatment is not guaranteed, and parents struggled with spiralling prescription and appointment costs:

*I have to apply for the long-term illness payment. [Son’s] medication bills were €200 a month. It’s supposed to be about €140, that’s a lot of money every month…* (Parent 34)

Parents can take ‘Carer’s Leave’ for up to two years (an employment break to care for relatives, with a small financial stipend). Some parents felt they had no other choice:

*I gave up working. She was diagnosed at three and I never went back...* (Parent 7)

HCPs asserted that high costs deterred treatment, as parents struggled to pay for everything:

*You might say ‘Oh, the dietitian might also be good’. But they pay extra or pay separately, so they pick and choose what they think is important* (HCP 1)

Adolescents did not discuss the costs of JIA treatment.

d) ***Issues with Methotrexate***

Adherence issues were discussed specifically for Methotrexate, a disease-modifying anti-rheumatic drug (DMARD). For adolescents, the unpleasant side-effects made them reluctant:

*I’m tired every day. I’m not a morning person as it is, but the methotrexate makes it ten times worse* (Adolescent 18)

Parents felt that long-term concerns also factored into this reluctance:

*She had a flare a while back; they had to increase the methotrexate and she was devastated. She said to me, “How am I ever going to get pregnant?”* (Parent 19)

Adolescent reluctance may also be related to alcohol consumption, which is contra-indicated while Methotrexate is prescribed. Irish adolescents commonly drink alcohol at social gatherings, so those with JIA would feel “different” from peers, according to parents and HCPs:

*That group between fourteen and sixteen, we have the biggest issues with... Maybe they can't drink and have Methotrexate… There's pressure on teenagers to drink* (HCP 9)

While avoiding Methotrexate is not exclusive to Irish adolescents, participants felt that avoidance in Irish adolescents may have been based on alcohol due to cultural practices around celebrating milestones; e.g., the ‘Junior Certificate’ (state exams taken midway through high school) are completed aged 14–15, and celebrations often include parties and alcohol, which may exacerbate feeling “different” in those taking Methotrexate.

#### 4. Views on web-based approach to self-management

The adolescents were overwhelmingly positive about the *TTC* website:

*I’ve never seen anything like it before. I’ve never seen a website that has all the information that you could think of* (Adolescent 7)

Parents felt it would be particularly useful when families were first diagnosed:

*For newly-diagnosed parents, who are finding their way in the first year, who don’t know what an appointment with an OT or a physio is…* (Parent 7)

They also praised the fact that the website could reassure adolescents on topics deemed too sensitive to discuss with parents:

*It’s good because 17A recently brought up about a tattoo when she’s older. There’s going to be something else that she wants to do that she won’t ask her mother… But now there’s a website where she can look* (Parent 17)

HCPs appreciated the availability of a trusted site with accurate information:

*It’s very comprehensive, but simplistic enough to follow. I was pretty impressed. I learned loads of stuff myself, because it’s a one-stop shop!* (HCP 2)

However, they worried that the website would replace traditional medical advice:

*My concern with that is they’ll go to the website before they go to the GP* (HCP 23)

Each participant group offered suggestions to make the *TTC* programme relevant for Irish users. Adolescents suggested Irish content: e.g., changing the names of medications and treatments (if trade names differed); replacing Canadian sports references with Irish sports; and adding video-clips featuring Irish adolescents:

*It would be good to have a mix of videos from different places, because people would know that it happens everywhere* (Adolescent 8)

As parents mainly used Google and North American websites for information, they proposed adding curated information suitable for Irish parents. Parents also wanted practical information regarding service access and entitlements:

*What you’re entitled to from the government... Links of where to go and how to apply. Services in their areas, walking groups and stuff* (Parent 15)

One HCP suggested that the instructional videos could link to existing videos online:

*You could have links to YouTube stretching programmes or how to perform certain exercises Then they could YouTube ‘hamstring stretches’ or ‘back stretches’* (HCP 20)

#### 5. Gaining understanding through social support

Adolescents spoke positively about JIA support organisations and meeting each other at events:

*I went on [organisation’s] road trip and met loads of new friends…I talk to [14A] all the time because she understands what I’m going through…* (Adolescent 16)

However, they avoided discussing their condition with non-JIA friends to not appear ‘different’:

*I’m sure I could talk to them, but I don’t. I stick to myself* (Adolescent 15)

This was confirmed by parents:

*I don’t think [19A] was talking about her condition with her friends. I’m not sure that she shares with them how she’s feeling every day* (Parent 19)

Parents saw arthritis organisations as vital sources of social support:

*That’s why I joined iCAN. I could interact on my own terms* (Parent 10)

*It’s tough going, if I wasn’t with these [other parents], I wouldn’t be sane* (Parent 3)

Support from school teachers varied. Some adolescents experienced understanding from teachers, while others felt singled out, making them feel different from classmates:

*[14A] and I go to the same school, so our teachers are pretty understanding. If I miss a day, I can catch up on work and print off the sheets and turn them in* (Adolescent 16)

*It’s things like [17A]‘s school tour. The teacher makes her sit at the front, and she’s like ‘No! Everyone will know there’s something wrong with me!*’ (Parent 17)

Parents and adolescents agreed that teachers are not fully equipped to understand JIA:

*The teacher said “Oh, [8A] is great, but he’s a bit slower than the others”. I said, ‘[8A] has arthritis’. And she said, ‘Does he? You’d never know it to look at him’.* (Parent 8)

Throughout the interviews, adolescents and parents highlighted social support from others living with JIA, and the idea was raised of integrating a peer-mentoring scheme, *iPeer2Peer,* into the *TTC* self-management program [26]. Adolescents liked discussing their condition with someone who understood:

*It’s harder to talk to someone who doesn’t have [JIA] than someone who’s gone through what you’ve gone through, who knows what mind-set you have* (Adolescent 8)

*Put [iPeer2Peer] on the front of the website so people will see it every time they log on. If they didn’t pick it the first time, they might a few weeks later* (Adolescent 14)

Parents were enthusiastic about their child linking up with older adolescents with JIA:

*I do think it’s a great idea!* (Parent 19)

HCPs spoke positively of peer-to-peer mentoring, but worried about the spread of misinformation:

*I would worry about life experiences they might share. If they had negative experiences with a staff member that would impact the mentee*. (HCP 33)

## Discussion

The identified themes match other countries, including the UK, US, and Canada [6, 22, 33], reinforcing that adolescents with JIA need information and support for their condition, to develop effective strategies to improve HRQL [6, 7, 10, 25]. Participants from all stakeholder groups agreed that Irish adolescents with JIA needed to become self-sufficient and take responsibility in managing their condition while transferring to adult care [17, 19, 34]. However, this independence can backfire when adolescents fail to adopt good self-care practices such as physiotherapy exercises or self-administering medications [35]. Methotrexate is often a contentious issue, partly due to contra-indications with alcohol, as previously mentioned by older adolescents in the UK [20]. Irish adolescents taking methotrexate may have felt hesitant to ‘stand out’ from their peers, according to parents and HCPs. Though underage drinking is declining across Europe, including Ireland, participants perceived that cultural practices involving alcohol meant Irish adolescents still drink more excessively than counterparts; this is partly corroborated by recent data [36]. Conversely, Canadian research found that adolescents sometimes used their JIA as a convenient means to avoid peer-pressure for risky behaviours such as underage drinking or unsafe sex [22].

Irish adolescents experienced lengthy wait-times for paediatric rheumatology consultations, despite guidelines stating that delays in diagnosis adversely impact disease prognosis, and should not exceed 10 weeks from symptom onset [37], as early diagnosis is crucial for interventions to successfully improve or maintain high levels of HRQL [13, 14] Almost 900 Irish children and adolescents are awaiting rheumatology services, and nearly 60% have been waiting for over one year [38, 39]. Several adolescents in this study quoted waiting two years for initial appointments and waiting times are increasing annually [39–41].

Regarding self-management, parents prioritised emotional and financial struggles, while adolescents focused on pain-management. The adolescents paced themselves to avoid injury or gave up cherished hobbies to reduce pain and discomfort. This resourcefulness has featured in previous qualitative studies on living with JIA, as adolescents ‘strive for normality’ [6, 20, 34], and persevere through pain to keep up with peers, rather than avoiding activities and being ‘different’ [6]. Adolescents may need to adjust coping strategies, and accept limitations as part of managing their condition [15]. Managing JIA was also difficult for parents, who struggled to support and advocate for their child; this struggle to ‘master’ their child’s chronic pain has been previously reported [35]. Arguably the most striking finding was the combative parental language when describing difficulties in accessing supports (*‘fight’; ‘battle’*). Similar language was used by parents of children with complex regional pain syndrome [35], but to our knowledge, has not emerged in arthritis research before. Such ‘*fighting’* exhausted parents, who relied on support from each other rather than seek formal psychological support for the emotional strain of JIA. The HCPs felt that stigma surrounding psychological services in Ireland deterred many families, echoed by the families themselves.

It is notable that families were wary of psychological services. A study of healthy Irish adults reported more favourable attitudes towards seeking psychological help than similar European counterparts [42]. However, as our study asked adolescents and adults with personal or professional experience of pain and associated emotional strains, their perspectives may differ. Indeed, interviews with Irish adults living with psychological illnesses revealed strong stigma and discrimination towards them, and a preference for disclosing to family and friends rather than health professionals [43], which reflects our findings. Future online interventions should address the emotional strain of living with chronic illness, to equip families with coping strategies, especially if reluctant to seek formal support.

In acquiring information, a clear discrepancy in perspectives emerged. Families often struggle for knowledge about JIA [6, 34], and while adolescents in this study had found information on JIA, others relied on their parents who felt the information from HCPs was inadequate. This conflicted with HCP perspectives, who were adamant that families received information at diagnosis and when new medications or treatments were introduced. Disagreements about knowledge-sharing were found previously in JIA research [44], and these discrepancies emphasise the need for accessible sources of information on JIA that are relevant for Irish families and allow them to share with HCPs.

The *TTC* website was accepted by all participant groups as a promising means of providing information. Adolescents and parents unanimously supported a centralised website to learn about JIA, reflecting previous findings [22, 29, 30], and future studies could compare the Irish experience of using self-management websites with different countries including UK and Canada. The HCPs appreciated that the website offers current information to adolescents between visits. However, one HCP was concerned that the website would replace GP visits. This could be addressed by incorporating messaging to refer families to their GP with concerns, as learning to navigate primary care is a key self-management skill during transfer to adult services. The HSE (Health Service Executive) in Ireland offers an interactive map of local services [45]; this could be embedded within the *TTC* website or linked to externally.

The adolescents revealed strong desires to “fit in”. Adolescents struggled with peers not understanding the restrictions of JIA, finding support instead from others with JIA with whom they identified more easily [20, 22]. Irish JIA adolescents often met through arthritis organisations and connected through social media. Internet-based initiatives are a promising means to promote ‘normality’ amongst adolescents with JIA [6, 21, 29], and this group strongly endorsed online peer-mentoring programmes like *iPeer2Peer* [26] to discuss their experiences with like-minded peers. In a systematic review of peer relationships in adolescents with chronic pain, parents reported their children were less socially-active and had fewer ‘close’ friends than healthy peers [11]. While this was not expressed here, the parents praised *iPeer2Peer* for allowing their child to socialise with similar adolescents. One HCP was concerned that adolescents would share bad experiences with each other; while this is possible, the benefits of online support groups outweigh the downsides [22, 46]. Ultimately, *iPeer2Peer* was regarded as a positive addition to the tailored self-management offered by *TTC*.

### Strengths

This study presents diverse viewpoints from adolescents with JIA across Ireland, their parents and their healthcare providers. Parents were exceptionally keen to participate, as some mentioned being ‘desperate’ for assistance. HCPs agreed to share their experience of treating adolescents with JIA in Ireland, despite busy schedules. A higher-than-average proportion of HCPs worked within a multi-disciplinary team as paediatric rheumatology services are geographically-centralised in Dublin and specialists worked together to treat young people with JIA. While this may be atypical, it produced a rich variety of viewpoints which we feel benefitted this study.

### Limitations

As with any qualitative study, the findings may apply only to that population and may not be generalisable. The distribution of JIA subtypes was skewed in our sample; as each subtype has different symptoms, treatment needs and their experiences with JIA may vary. Larger samples (with a better distribution of subtypes) would offer stronger conclusions. Of further note is the lack of participation by male adolescents and parents: our sample was predominantly female, and the views shared may not apply equally to boys with JIA and their fathers. All participating families were affiliated with national arthritis organisations, but families outside of this network may have different experiences with JIA, and these perspectives should be included. Finally, the busy schedules of the paediatric rheumatology consultants limited interviews to brief discussions, where in-depth discussions would have provided stronger insight.

## Conclusion

Discrepancies in the availability and quality of arthritis information demonstrate the need for a concise source of information on JIA self-management [20, 24]. While general information is available on developing self-management skills, there is scope for providing easily-accessible, accurate information on JIA to Irish families. The TTC website was well-received by Irish participants, with generally positive reaction for combining the online self-management program with Skype-based peer-mentoring [26] for JIA adolescents to share their experiences. In short, the acceptability of adapting an existing self-management intervention for Irish users has been confirmed.

## Abbreviations

HCP: Healthcare professional
HRQL: Health-related quality-of-life
HSE: Health Service Executive
JIA: Juvenile idiopathic arthritis
TTC: “Teens Taking Charge”
